# Antimicrobial Activity of Bioactive Peptides on Resistant Enterobacteriaceae and the Viability of *Giardia duodenalis* Cysts Isolated from Healthy Dogs

**DOI:** 10.3390/vetsci13010044

**Published:** 2026-01-03

**Authors:** Antonio Santaniello, Emanuela Roscetto, Umberto Galdiero, Paola Pepe, Antonio Bosco, Ida Boccino, Ludovico Dipineto, Maria Rosaria Catania, Paolo Grieco

**Affiliations:** 1 Department of Veterinary Medicine and Animal Production, University of Naples Federico II, 80137 Naples, Italy; antonio.santaniello2@unina.it (A.S.); ludovico.dipineto@unina.it (L.D.); 2 Department of Molecular Medicine and Medical Biotechnology, University of Naples Federico II, 80131 Naples, Italy; emanuela.roscetto@unina.it (E.R.); umberto.galdiero@unina.it (U.G.); 3 Centro Regionale di Monitoraggio delle Infezioni Parassitarie, Department of Veterinary Medicine and Animal Production, University of Naples Federico II, 80137 Naples, Italy; paola.pepe@unina.it (P.P.); antonio.bosco@unina.it (A.B.); 4 Department of Pharmacy, University of Naples Federico II, 80131 Naples, Italy; ida.boccino@unina.it (I.B.); paolo.grieco@unina.it (P.G.)

**Keywords:** AMR, *Enterobacter* spp., *Giardia*, one health, zoonosis, dogs

## Abstract

*Enterobacter* spp. and *Giardia duodenalis* are two resistant and globally distributed pathogens widely reported in humans and animals worldwide. To contrast the antimicrobial resistance phenomenon, this study aimed to evaluate the in vitro activity of antimicrobial peptides (AMPs), such as temporins, on the viability of *Enterobacter* spp. and *G. duodenalis* cysts isolated from naturally infected dogs. A total of 50 clinically healthy dogs were sampled, with 25 in each of the two sampling sessions. Rectal and oral swabs for *Enterobacter* spp. and fecal samples for *G. duodenalis* isolation were carried out. The AMPs were synthesized by a solid-phase peptide synthesis approach. *Enterobacter ludwigii* and *E. hormaechei* strains were isolated from two different dogs, while 16 dogs were positive for *G. duodenalis*. Subsequently, cysts and bacterial strains were treated with the following peptides: TL-34, TL-48, TL-42, TL-51, RB-71, and RB-58 at different concentrations (i.e., 250 mg/mL, 125 mg/mL, 62. mg/mL, and 31.25 for *Enterobacter* spp. strains and 0.15 mg/mL, 0.3 mg/mL, 0.6 mg/mL, and 1.2 mg/mL for *G. duodenalis* cysts). The results showed antimicrobial activity of TL-34, TL-48, and RB-71 against the two *Enterobacter* strains, while TL-51/TL-42 and RB-58 were not effective. In contrast, for *G. duodenalis,* TL-34, TL-48, TL-42, and RB-58 were effective at the two highest concentrations, inhibiting the viability of 100% of *Giardia* cysts, while the other two peptides (TL-51 and RB-71) showed less effectiveness. The AMPs used in this study have in vitro antimicrobial activity on cysts and bacterial strains and appear to have potential for the treatment of these infections in dogs.

## Simple Summary

Dogs can carry microorganisms that may cause illness in animals and people, including certain bacteria and intestinal protozoa. Some of these microorganisms are becoming difficult to treat because they no longer respond well to common drugs. For this reason, new ways to control infections must be sought urgently. In this study, we examined whether small natural-like peptides called temporins could reduce or stop the growth of these germs. We collected samples from fifty dogs and selected the samples positive for *Enterobacter* strains and the intestinal protozoon (*Giardia*). We exposed the bacteria and the parasite to six different peptides. Three of these peptides inhibited the growth of the bacteria, while four were able to completely prevent the survival of the *Giardia* parasite, although their effectiveness depended on how much of the peptide was used. These findings suggest that certain peptide molecules may offer new options for controlling infections in dogs, especially at a time when resistance to current medicines is increasing. Further research may help determine how these peptides could be used safely in real-world treatments.

## 1. Introduction

Zoonotic infections are a major global threat to public and animal health and welfare. Intestinal bacteria and protozoa infecting animals are responsible for numerous human foodborne, contact, and waterborne infections, becoming very severe and even fatal [1]. These diseases pose a significant public health challenge, as they can lead to epidemic outbreaks and have significant repercussions on human populations and ecosystems [2]. Although zoonotic viruses are the main cause of major health crises, bacteria and parasites also pose a significant threat to the large-scale spread of zoonoses [3]. Several zoonotic diseases have been associated with multidrug-resistant bacteria responsible for nosocomial deaths, such as *Enterobacter* spp., and intestinal protozoa, such as *Giardia duodenalis*, recognized by WHO in their “priority pathogens list” [4].

*Enterobacter* spp. is an opportunistic pathogen frequently isolated in human and animal populations, in hospital environments, and in urban areas where fecal contamination represents a significant yet often underestimated risk factor for public health [5,6]. Transmission between humans and animals occurs through direct or indirect contact of their mucosal surfaces with the host organism [7]. Particularly, *Enterobacter hormaechei*, belonging to the *Enterobacter cloacae* complex, has been isolated in various clinical contexts, such as cases of bronchitis in calves, and represents a growing concern due to both intrinsic and acquired resistance, including ESBL and carbapenemase production [8]. Regarding *Enterobacter ludwigii*, veterinary data are more limited, but by analogy with the human context, where it is associated with catheter-related infections, it may act as an opportunistic pathogen in similar situations in animals [9,10].

*Giardia duodenalis* (syn. *G. lamblia* and *G. intestinalis*) is a protozoan parasite of the intestinal tract that infects humans as well as domestic and wild animals. It causes giardiasis, a disease of major worldwide public health relevance [11]. Asymptomatic cases are frequent; however, after the incubation phase, infected individuals can experience various symptoms, including diarrhea, bloating, epigastric pain, nausea, and vomiting [12]. Even with the growing recognition of the significant morbidity linked to *Giardia* infections, treatment options still rely on a limited range of broad-spectrum antimicrobials. Rising resistance and treatment failures further undermine the effectiveness of frontline drugs [13]. *Giardia intestinalis* exhibits marked variability in its susceptibility to routinely used antigiardial compounds, including metronidazole, albendazole, fenbendazole, and nitazoxanide, under both in vitro and in vivo conditions [14]. Over the past two decades, several in vitro drug-susceptibility assays have been established, primarily based on axenic trophozoite cultures and viability or growth inhibition endpoints. These systems consistently demonstrate that reduced susceptibility and even stable resistance can arise, particularly to 5-nitroimidazoles such as metronidazole [15].

More recently, methodological advances have enabled the development and refinement of in vitro assays specifically designed to assess the impact of drugs or alternative compounds on the viability and structural integrity of *Giardia* cysts, providing promising and reproducible results [16].

Antimicrobial resistance (AMR) is a serious global concern compromising the success of treatment in bacterial infections both in humans and in animals, resulting in a lack of effective antibiotics and high medical expenses [17]. Bacteria belonging to the *Enterobacteriaceae* family are the most concerning, where multidrug resistance is constantly increasing [18]. The decreased availability or ineffectiveness of antibiotics causes prolonged hospital stays, increased duration and severity of infections, increased risk of infection transmission among the hospital population, and, finally, increased mortality [4,19]. Due to the AMR phenomenon, the discovery of novel molecules as an alternative strategy to antibiotics represents a high-priority research goal, alongside regular measures of surveillance and control [20,21,22,23].

Antimicrobial peptides (AMPs) could provide a valid opportunity to overcome and control AMR [22]. These AMPs are promising candidates for a new generation of anti-infective agents in both human and veterinary medicine. AMPs are short and generally positively charged peptides [23,24]. Many of them can kill microbial pathogens directly, whereas others act indirectly by modulating the host’s defensive systems [25,26,27].

Amphibians represent one of the most abundant sources of AMPs (bombinins, brevinins, esculentins, temporins), which are present in the serous glands of the skin and secreted holocrinely in response to stress or cellular damage. The AMPs characterized to date include several classes with distinct structural and functional properties. AMPs can be classified according to various criteria, such as their biological targets, source organisms, physicochemical properties, biosynthetic pathways, structural features, covalent bonding patterns, and predominant antibacterial activity. Structural classification based on three-dimensional conformation is one of the most widely used approaches among these. According to their secondary and tertiary structures, AMPs fall into one of four categories: α, β, α–β, and non-α–β. These classes are defined by the presence of α-helical structures, β-sheet motifs, a combination of α-helices and β-sheets, or the absence of both α-helical and β-sheet structural elements, respectively. AMPs exhibit a variety of functional properties, including antibacterial, antiviral, anticancer, anti-inflammatory, and antioxidant properties [28,29].

Temporins were first identified in 1996 and are produced by dermal glands located mainly in the dorsal region of the common frog *Rana temporaria*, from which they take their name [22]. Structurally, these AMPs are short peptides composed of 10–14 residues, which carry a net positive charge at neutral pH and adopt an amphipathic α-helical structure when in contact with membranes or within hydrophobic environments [30]. Temporins are known to be particularly active against Gram-positive bacteria, with minimal inhibitory concentrations (MIC) ranging from 2.5 to 20 µM [31,32]. In particular, the isoform L (temporin L, TL), Phe-Val-Gln-Trp-Phe-Ser-Lys-Phe-Leu-Gly-Arg-Ile-Leu-NH_2_, is also active against Gram-negative bacteria and yeast strains [33,34].

Therefore, this study aims to evaluate the efficacy of novel anti-infective compounds, such as temporin L derivatives, against multidrug-resistant *Enterobacter* spp. and the enteric protozoan *Giardia duodenalis* in potentially infected shelter dogs.

## 2. Materials and Methods

### 2.1. Sampling

The study was conducted in a single animal shelter in Southern Italy and included two sampling sessions carried out between December 2023 and July 2024. In each session, 25 dogs, apparently in good health (as stated by the owner of the kennel), well-nourished, and without having received antibiotic treatments in the last month, were randomly selected from the 200 dogs housed in the facility. For each dog, the age, sex, microchip number, and cage number were recorded, and a pair of swabs (oral and rectal) in Amies transport medium, along with fecal samples, were collected in order to detect *Enterobacter* spp. and *G. duodenalis*, respectively. All procedures were performed in accordance with animal welfare guidelines. Subsequently, antimicrobial susceptibility testing was carried out on *Enterobacter* isolates.

### 2.2. Antimicrobial Peptides

As described by Buommino et al. [34], the peptides used in the present study (shown in Table 1) were synthesized using standard solid-phase peptide synthesis (SPPS), applying the dominant Fmoc/tBu orthogonal protection strategy. This consists of cycles of coupling and deprotection steps by employing the base-labile, N-alpha-Fmoc protecting group, along with acid-labile side-chain protections and peptide–resin linkage. The incorporation of cross-links carried out in the solid phase and for each compound were applied using a specific synthetic strategy. Finally, all the peptides were purified by HPLC and characterized by Electrospray Ionization–Mass Spectrometry (ESI-MS) and Nuclear Magnetic Resonance (NMR) spectroscopy.

### 2.3. Bacterial Isolation and Identification

All collected swabs were transported to the Microbiology Laboratory of Veterinary Medicine and Animal Production of Naples, Italy, and processed according to the standard operating procedure for *Enterobacter* spp. In detail, each sample was placed in pre-enrichment broth (peptone water; PW; Oxoid, Rodano, Italy) and incubated aerobically at 37 °C for 24 h. Subsequently, the samples were streaked onto selective and differential media. An aliquot of broth culture (10 µL) was seeded onto MacConkey Agar medium (Oxoid, Rodano, Italy) and incubated at 37 °C for 24 h. All colonies were subjected to morphological recognition, oxidase and catalase tests, and streaked onto chromogenic media, such as Tryptone Bile X-Gluc agar (TBX; Oxoid, Rodano, Italy) at 37 °C for 24 h. In addition, bacterial identification was conducted by assessing the growth status on CHROMagar orientation medium [35] using the API 20E kit (SYSMEX bioMérieux Co., Ltd., Tokyo, Japan) and confirmed by Matrix-Assisted Laser Desorption/Ionization Time-Of-Flight (MALDI-TOF) analysis with the Bruker MALDI Bio-typer system (Bruker Daltonics, Bremen, Germany).

### 2.4. Giardia Cyst Isolation and Identification

Each fecal sample was analyzed by the Mini-FLOTAC technique with an analytic sensitivity of five cysts per gram (CPG) of feces, using a flotation solution based on zinc sulfate (s.g. = 1.35) to detect *G. duodenalis* cysts [36]. The positive samples with a mean value of 15,000 CPG for *G. duodenalis* were processed to purify the cysts for the in vitro tests using the recovery technique reported in Morgoglione et al. [37]. Briefly, the fecal samples were homogenized and filtered under running water through sieves with different mesh sizes, as follows: 1 mm, 250 μm, 100 μm, 50 μm, and 25 μm for *G. duodenalis* cysts. The fecal suspension obtained was centrifuged for three minutes at 170 g, and the supernatant was discarded. Finally, the pellets were resuspended with 40% sucrose solution and transferred to new tubes. To obtain a clear aqueous solution with *Giardia* cysts, two successive rounds of centrifugation with distilled water were performed. After thorough homogenization of the suspension into the tubes, ten aliquots of 10 μL each were taken to count the number of *Giardia* cysts at 100× and 400× magnifications. To assess the *Giardia* assemblages from the naturally infected dogs, molecular studies were performed as described by Ciuca et al. [38].

### 2.5. Antimicrobial Susceptibility Test

The antibiotic susceptibility profile of the strains was assessed using the disk diffusion method following the Kirby–Bauer procedure, in accordance with the guidelines of the Clinical and Laboratory Standards Institute (CLSI) [39]. Briefly, bacterial colonies (18 h of growth) were suspended in sterile physiological saline solution to reach a turbidity equivalent to the 0.5 McFarland standard (approximately 1–2 × 10^8^ CFU/mL). This suspension was uniformly spread onto Mueller–Hinton agar plates and antibiotic-impregnated disks were then placed on the agar surface. The plates were incubated at 37 °C for 24 h under standard aerobic conditions.

Specifically, the following antibiotics (Liofilchem, Roseto degli Abruzzi, Italy) were tested: Amoxicillin–Clavulanic Acid (AUG, 30 μg); Ceftriaxone (CRO, 30 μg); Aztreonam (ATM, 30 μg); Ceftazidime–Clavulanic Acid (CTLC, 40 μg); Gentamicin (CN, 10 μg); Nalidixic Acid (NA, 30 μg); Tetracycline (TE, 30 µg); Ciprofloxacin (CIP, 5 μg), Enrofloxacin (ENR, 5 μg); Norfloxacin (NOR, 5 μg); Piperacillin (PRL, 30 μg); Sulfatrimetropin (SXT, 30 μg); Chloramphenicol (C, 30 µg); Imipenem (IMI, 10 μg). *Enterobacter hormaechei* ATCC 700323 was used as the susceptible reference strain in each experiment.

After incubation, the diameters of the inhibition zones around the disks were measured in millimeters, and the results were interpreted according to CLSI guidelines [39].

### 2.6. Antimicrobial Activity Assays of Peptides

#### 2.6.1. Determination of the Minimum Inhibitory Concentration (MIC) and the Minimum Bactericidal Concentration (MBC)

The antimicrobial properties of temporins were evaluated using a broth microdilution method in 96-well microtiter plates. Bacterial inocula were prepared and adjusted to a turbidity equivalent to the 0.5 McFarland standard. The suspensions were then diluted 1:100 in Mueller–Hinton broth (MHB), and a 100 μL aliquot was added to each well.

Temporins were prepared in two-fold serial dilutions in MHB broth, with final concentrations ranging from 250 μg/mL to 0.48 μg/mL. An equal volume (100 μL) of each peptide dilution was added to the wells containing the inoculum, resulting in a final volume of 200 μL per well. Wells without peptide treatment were used as negative controls.

The plates were incubated under shaking at 37 °C overnight under aerobic conditions. Bacterial growth was quantified by measuring the optical density at 590 nm using a microplate reader, and the percentage of growth inhibition was calculated by comparing the absorbance values obtained with those of the positive controls. MIC and MBC values were calculated. MIC was defined as the lowest peptide concentration at which no bacterial growth was detected. To determine the MBC, aliquots from wells with no growth were plated onto blood agar and incubated under the same conditions previously described. The MBC was identified as the lowest concentration of peptide that resulted in complete eradication of viable bacteria, as indicated by the absence of colony formation. All conditions were tested in triplicate and twice to ensure reproducibility.

#### 2.6.2. Antimicrobial Activity on the Viability of Giardia Cysts

Isolated cysts were treated with six temporins (TL-34, TL-48, TL-42, TL-51, RB-71, and RB-58) at different concentrations (0.15 mg/mL, 0.3 mg/mL, 0.6 mg/mL, and 1.2 mg/mL), metronidazole (50 μg/mL) (positive control) and phosphate-buffered saline (PBS 1%) (negative control) for different contact times (30, 60, and 180 min) [37]. The viability of the cysts was evaluated by staining with 1% eosin dye following the method described by Dehghani-Samani et al. [40] with some modifications. Fifteen minutes after exposure to the dye, the unstained cysts (which did not absorb the dye) were considered potentially viable, and the stained cysts were considered non-viable.

The percentage of non-viable cysts was calculated using the following formula:

(1 − (total number of viable cysts per mL of aliquot/total number of cysts per mL of aliquot)) × 100.

### 2.7. Statistical Analysis

To evaluate the viability of *Giardia* cysts, one-way ANOVA was performed to detect the significant difference between the treated and non-treated groups with Turkey’s post hoc tests. For all comparisons, a level of α = 0.05 was assumed, and the obtained *p*-values were rounded to two decimal places. Statistical analysis was performed using SPSS Statistics v.23 (IBM, Armonk, NY, USA).

## 3. Results

From a total of 100 swab samples, two *Enterobacter* strains were isolated and identified: *E. ludwigii* from an oral swab and *E. hormaechei* from a rectal swab. In addition, 16 of the screened dogs tested positive for *G. duodenalis*, and molecular analysis identified the assemblage C.

### 3.1. Antibiotic Susceptibility Profiles of Isolates

The antibiotic susceptibility profiles of the *Enterobacter* species involved in the study were obtained using the Kirby–Bauer test and are presented in Table 2. The strains were found to be resistant to several of the 14 tested antibiotics. Indeed, *E. ludwigii* was only susceptible to gentamicin, while the clinical *E. hormaechei* was also susceptible to chloramphenicol and norfloxacin. The reference strain ATCC 700323, in contrast, was sensitive to most of the antibiotics tested except for ceftazidime–clavulanic acid and aztreonam.

### 3.2. Antibacterial Activity of Temporins

The antibacterial activity of the tested temporins was evaluated against *Enterobacter* strains isolated from dogs and reference strain *E. hormaechei* ATCC700323. The peptides were found to be active against all tested strains, and the MIC values are reported in Table 3. TL-34 and TL-48 were active at 125 µg/mL, showing bactericidal effects (MIC = MBC) against the clinical *Enterobacter* isolates. Regarding the ATCC 700323 reference strain, TL-34 exhibited a MIC value at 15 µg/mL and TL-48 at 62.5 µg/mL, with MBC values at 125 µg/mL and 62.5 µg/mL, respectively. RB-71 displayed bactericidal activity (MIC = MBC) up to 31 µg/mL against *E. ludwigii* and up to 62.5 µg/mL against both *E. hormaechei* strains. In contrast, TL-51/TL-42 and RB-58 showed no activity against any of the strains tested.

### 3.3. Activity of Temporins on Giardia Cyst Viability

The results of the study demonstrate a clear dose- and time-dependent effect of the tested temporins on the viability of *Giardia* cysts. In particular, at the two highest concentrations and after 180 min of exposure, four of the tested temporins—TL-34, TL-48, TL-42, and RB-58—completely inhibited cyst viability (100%), with a statistically significant difference (*p* < 0.001) compared to the control groups.

The remaining two temporins, TL-51 and RB-71, exhibited comparatively lower efficacy. Although their inhibitory effects were not negligible, complete inhibition of cyst viability (100%) was achieved only at the highest tested concentration and following prolonged exposure of 180 min (Table 4).

## 4. Discussion

The gastrointestinal tract is a major reservoir for the emergence and selection of antibiotic-resistant bacteria in humans, companion animals, and farm animals. Indeed, in this context, species diversity and abundance are essential conditions for horizontal genetic exchange. Under favorable conditions, intestinal bacteria, particularly *Enterobacterales*, can translocate to other sites and cause opportunistic infections in previously healthy individuals and animals. In veterinary hospitals, as well as in those serving humans, Gram-negative bacteria of the gut microbiota represent a growing cause of difficult-to-treat infections [41]. Furthermore, pets, especially dogs, share domestic environments with humans, and their close contact with owners offers opportunities for the transmission of resistant bacteria to humans through direct or indirect contact.

Within the genus *Enterobacter*, some species have been associated with nosocomial infections and epidemics [42,43,44]. *Enterobacter* spp. pathogenicity and virulence factors are not well understood due to limited studies. They secrete enterotoxins, alpha-hemolysins, and cytotoxins and can acquire plasmids carrying virulence and resistance genes [45]. *Enterobacter* spp. are currently also included in the multidrug-resistant ESKAPE pathogens (*Enterococcus faecium*, *Staphylococcus aureus*, *Klebsiella pneumoniae*, *Acinetobacter baumannii*, *Pseudomonas aeruginosa*, and *Enterobacter* spp.) and represent a major cause of infections in hospital environments [4,45]. A recent review and meta-analysis by Santaniello et al. [46] highlighted the presence of ESKAPE group bacteria, in particular *Enterobacter* spp., in dogs, reporting epidemiological investigation data showing its presence mainly in urine samples, intravenous catheters, and ocular, oral, and rectal swabs.

The mechanisms and genes underlying antibiotic resistance in *Enterobacter* and its clinical implications have been extensively studied [47,48,49]. *Enterobacter* spp. has intrinsic resistance to ampicillin, amoxicillin, first-generation cephalosporins, and cefoxitin due to the expression of a constitutive AmpC β-lactamase, making treatment a challenge [42,50].

AMPs, key components of the innate immune system, are typically cationic at physiological pH due to the presence of basic amino acid residues (e.g., arginine and lysine) and display amphipathic structures driven by hydrophobic residues. This unique combination of properties facilitates their interaction with the negatively charged membranes of microorganisms, leading to membrane insertion, destabilization, and disruption. In addition to membrane alterations, AMPs can also target intracellular components, enhancing their antimicrobial potential. Because their primary mechanism of action targets the cell membrane—a structure that is difficult for microorganisms to modify without compromising viability—the development of resistance to AMPs is considered less likely.

On this basis, AMPs are the subject of many studies as highly promising candidates for future antimicrobial therapies. Di Somma et al. [47] showed that temporin L inhibited the cell division mechanism of *E. coli.* Ji F. et al. [50] detected the antimicrobial activity of temporin-1CEc against multidrug-resistant *Staphylococcus epidermidis*. Roscetto et al. [51] demonstrated the antimicrobial activity of two temporin L analogs, peptides 1B and C, against carbapenemase-producing clinical isolates of *Klebsiella pneumoniae*. Furthermore, the antifungal properties of acylated derivatives of temporin L against drug-resistant *Candida albicans* strains were also shown [30].

The results obtained in this study show significant differences between the antimicrobial activities of the tested temporins. TL-34, TL-48, and RB-71 showed effective bactericidal activity against all tested bacterial strains, while TL-42, TL-51, and RB-58 showed no activity. RB-71 proved to be the most potent, achieving bactericidal activity at lower concentrations than the other peptides. These data indicate that temporins may maintain efficacy even against multidrug-resistant strains, offering a potential advantage over conventional antibiotics, in line with previous studies on temporin L peptides and analogs [26,27,28,29,30,51,52].

With regard to *G. duodenalis*, our data show a clear dose- and time-dependent effect. Four temporins (TL-34, TL-48, TL-42, and RB-58) completely inhibited cyst viability (100%) at the two highest concentrations after 180 min, while TL-51 and RB-71 showed moderate activity, achieving 100% inactivation only at the maximum concentration and after prolonged exposure. This result is particularly relevant since many previous studies have focused on trophozoites, while cyst treatment is crucial for limiting transmission [52,53].

The complete inactivation of cysts observed over four time periods suggests that the structure and physicochemical properties of peptides strongly influence their efficacy, probably through interactions with the membrane or alterations in essential metabolic processes [52,53,54]. Furthermore, the findings are consistent with data, indicating that the intestinal epithelium can activate endogenous antimicrobial peptides in response to *Giardia* infections, suggesting a complementary mechanism between natural defense and synthetic temporins [54].

Research on AMPs directed against protozoan parasites is still emerging, though several key studies provide useful benchmarks. For example, Aley et al. [55] demonstrated that cryptdins and other cationic neutrophil peptides effectively killed *G. duodenalis* trophozoites in vitro, with clear dose- and time-dependence.

More recently, a review [53] highlighted the broad potential of AMPs against protozoan infections, including giardiasis, emphasizing how sequence, charge, hydrophobicity, and structure influence potency and selectivity. The results of the present study are in line with these previous observations: first, we also found a clear dose/time effect (higher concentrations + prolonged exposure = greater efficacy); second, the fact that not all peptides were equally effective further reflects the importance of temporin physicochemical properties. The most novel finding in this study is the demonstration of complete cyst inactivation (100% loss of viability) for four temporins under defined conditions, which goes beyond many previous trophozoite-based assays.

Furthermore, the literature on host-derived AMPs and *Giardia* demonstrates that the intestinal epithelium up-regulates defensins and other AMPs in response to *Giardia* infection [53], suggesting that natural peptide-mediated defense plays a role in vivo. The present study can be viewed as a complementary synthetic peptide approach that may mimic or extend these natural defense mechanisms.

Moreover, the findings of this study highlight the value of in vitro assays as preliminary screening tools for identifying compounds with potential antiprotozoal activity against *G. duodenalis*. Such assays, when based on the standardized viability of *Giardia* cysts, provide an efficient first step to narrow down promising candidates prior to in vivo evaluation [14,15]. However, only in vivo studies can ultimately confirm the therapeutic efficacy, pharmacodynamic relevance, and clinical applicability of the tested products, given the well-known discrepancies that may occur between in vitro susceptibility and real treatment outcomes [55]. The clinical use of these new compounds may still take time, as it is necessary to optimize the formulation of the AMPs to make them stable for oral or injectable use while maintaining their specificity and efficacy [29].

Our findings provide important insights, extending the potential of temporins against *Enterobacter* strains and *G. duodenalis*. Future studies on larger populations will certainly be necessary to obtain more meaningful results and estimates on the efficacy of temporins as an alternative therapeutic treatment.

## 5. Conclusions

The growing prevalence of antimicrobial resistance increases the need for therapeutic alternatives to conventional antimicrobials, particularly for bacteria, such as those in the ESKAPE group, to which *Enterobacter* belongs. On the other hand, *Giardia intestinalis* exhibits marked variability in its susceptibility to commonly used anti-*Giardia* compounds. This study demonstrated the antimicrobial activity of temporin derivatives against two multidrug-resistant *Enterobacter* species and *Giardia duodenalis* cysts isolated from healthy dogs. Comparing the activity of various temporin L derivatives on *Enterobacter* and *Giardia* cysts, we found that RB71 was the most active analog against both *Enterobacter* species, while RB58 was the derivative with the greatest activity against *Giardia* cysts, although it was not effective against the tested bacteria. This result may be due to the different structure and complexity of the cell envelopes of the two target microorganisms. Indeed, *Enterobacter* is a Gram-negative bacterium with an outer membrane, while *Giardia* cysts are resistant structures with walls composed primarily of N-acetylgalactosamine polymers. In particular, the results regarding the activity of these derivatives on *Giardia duodenalis* cysts seem noteworthy, as the effect of temporins on *Giardia* remains poorly investigated. These results provide insights into the potential therapeutic applications of temporin analogs as antimicrobial agents, paving the way for future studies examining the in vivo efficacy of these compounds and optimizing their pharmacological properties.

## Figures and Tables

**Table 1:** Antimicrobial peptides based on the sequence of temporin L.

Acronym	Peptides	Sequence
**TL**	Temporin L	FVQWFSKFLGRIL-NH_2_
**TL-34**	Temporin L 34	FV**P**WFSKF**dL**GRIL-NH_2_
**TL-48**	Temporin L 48	FV**P**WFSKF**dLdK**RIL-NH_2_
**TL-42**	Temporin L 42	FV**P**WFSKF**dLdP**RIL-NH_2_
**TL-51**	Temporin L 51	FV**P**WFSKF**dLX**RIL-NH_2_
**RB-71**	Temporin L 71	**Lipid-X**FV**P**WFSKFLKRIL-NH_2_
**RB-58**	Temporin L 58	FVPWF**[K**KFdL**E]**RIL-NH_2_

**Notes**: d = D-isomer of specific aminoacids; X = Aic, 2-aminoindane-2-carboxylic acid; RB-71 contains valeric acid at N-terminal sequence; RB-58 contains a lactam bridge.

**Table 2:** Phenotypic antibiotic resistance profiles of the isolated *Enterobacter* strains.

Strains	Antibiotics Tested													
	CIP	TE	CN	SXT	NA	CRO	CTLC	PRL	C	NOR	ENR	IMI	ATM	AUG
*E. hormaechei*	I	R	S	I	I	R	R	R	S	S	I	R	R	R
*E. ludwigii*	R	R	S	I	R	R	R	R	I	R	R	R	R	R
*E. hormaechei ATCC 700323*	S	S	S	S	S	S	R	S	S	S	S	S	R	S

**Legend.** S: susceptible; I: intermediate; R: resistant; CIP: Ciprofloxacin; TE Tetracycline; CN: Gentamicin; SXT: Sulfatrimethoprin; NA: Nalidixic Acid; CRO: Ceftriaxone; CTLC: Ceftazidime–Clavulanic acid; PRL: Piperacillin C: Chloramphenicol; NOR Norfloxacin; ENR: Enrofloxacin; IMI: Imipenem; ATM: Aztreonam; AUG: Amoxicillin–Clavulanic Acid.

**Table 3:** Antibacterial activity of tested temporins.

Strains	MIC Values (µg/mL) of Temporins					
	TL-34	TL-42	TL-48	TL-51	RB-58	RB-71
*E. hormaechei*	125	nd	125	nd	nd	62.5
*E. ludwigii*	125	nd	125	nd	nd	31
*E. hormaechei ATCC 700323*	15	nd	62.5	nd	nd	62.5

nd = not detected.

**Table 4:** The results of in vitro antimicrobial activity of bioactive temporins on the viability of *Giardia* cysts.

Bioactive Peptides	Time Exposure (Min)	Non-Viable Cysts (%)			
		Concentration (mg/mL)			
		0.15	0.3	0.6	1.2
TL-34	30	0.0	21.0	36.0	74.8
	60	69.2	65.2	81.4	89.5
	180	70.0	87.5	100.0	100.0
TL-48	30	21.0	22.20	36.0	78.0
	60	21.20	68.9	94.3	100.0
	180	50.0	75.0	100.0	100.0
TL-42	30	8.0	11.1	59.0	100.0
	60	12.5	66.7	78.0	100.0
	180	50.0	100.0	100.0	100.0
TL-51	30	25.5	33.30	50.0	77.40
	60	52.5	60.0	89.8	100.0
	180	80.0	93.0	98.8	100.0
RB-71	30	0.0	33.3	40.0	70.0
	60	11.2	26.7	53.0	73.3
	180	56.0	78.8	89.0	100.0
RB-58	30	0.0	25.0	33.3	42.5
	60	15.0	39.0	52.9	61.5
	180	86.0	100.0	100.0	100.0
Positive Control Metronidazole (50 μg/mL)	30	81.5			
	60	89.8			
	180	100.0			
Negative Control (PBS 1%)	30	0.0			
	60	0.0			
	180	0.0			

## Data Availability

The original contributions presented in this study are included in the article. Further inquiries can be directed to the corresponding author.

## Abbreviations

The following abbreviations are used in this manuscript:

AMP	Antimicrobial Peptides
AMR	Antimicrobial Resistance
MIC	Minimum Inhibitory Concentration
MBC	Minimum Bactericidal Concentration
